# Effects of cannabinoid receptor type 2 in respiratory syncytial virus infection in human subjects and mice

**DOI:** 10.1080/21505594.2017.1389369

**Published:** 2017-12-08

**Authors:** Alireza Tahamtan, Yazdan Samieipoor, Fatemeh Sadat Nayeri, Ali Akbar Rahbarimanesh, Anahita Izadi, Ali Rashidi-Nezhad, Masoumeh Tavakoli-Yaraki, Mohammad Farahmand, Louis Bont, Fazel Shokri, Talat Mokhatri-Azad, Vahid Salimi

**Affiliations:** a Department of Virology, School of Public Health, Tehran University of Medical Sciences, Tehran, Iran; b Fetal and Neonatal Research Center, Tehran University of Medical Sciences, Tehran, Iran; c Bahrami Children Hospital, Tehran University of Medical Sciences, Tehran, Iran; d Maternal, Fetal and Neonatal Research Center, Tehran University of Medical Sciences, Tehran, Iran; e Department of Biochemistry, Iran University of Medical Sciences, Tehran, Iran; f University Medical Center Utrecht (UMCU), Wilhelmina Children's Hospital (WKZ), Department of Pediatrics, Utrecht, The Netherlands; g Department of Immunology, School of Public Health, Tehran University of Medical Sciences, Tehran, Iran

**Keywords:** Acute respiratory tract infection, CB2 receptors, single nucleotide polymorphism, immunomodulation, Respiratory syncytial virus, respiratory viral infection

## Abstract

An accumulating body of evidence suggests that the endocannabinoid system plays a significant role in pathophysiological processes and impacts disease severity. Here we investigate the possible role of a cannabinoid receptor type 2 (CB2) functional variant in determining disease severity and the potential pharmacological therapeutic effects of CB2 activation in viral respiratory infection. The common missense variant (CAA/CGG; Q63R) of the gene-encoding CB2 receptor (CNR2) was evaluated in 90 inpatient and 90 outpatient children with acute respiratory tract infection (ARTI). The frequency distribution of respiratory syncytial virus (RSV)-the main cause of severe cases of bronchiolitis and pneumonia in children-was studied in all collected samples. The mechanism through which CB2 affects clinical outcomes in case of RSV infection was studied in Balb/c mice model using AM630 as a CB2 antagonist. The potential therapeutic effect of CB2 activation during RSV infection was studied using a selective agonist, JWH133. The CB2 Q63R variation was associated with increased risk of hospitalization in children with ARTI. Children carrying the QQ genotype were more prone to developing severe ARTI (OR = 3.275, 95% CI: 1.221–8.705; p = 0.019). Of all the children enrolled in the study, 83 patients (46.1%) were found positive for RSV infection. The associated risk of developing severe ARTI following RSV infection increased more than two-fold in children carrying the Q allele (OR = 2.148, 95% CI: 1.092–4.224; *p* = 0.026). In mice, the blockade of CB2 by AM630 during RSV infection enhanced the influx of BAL cells and production of cytokines/chemokines while exaggerating lung pathology. CB2 activation by JWH133 reduces the influx of BAL cells and production of cytokines/chemokines while alleviating lung pathology. Collectively, CB2 is associated with RSV severity during infancy and may serve as a therapeutic target in RSV infection through the alleviation of virus-associated immunopathology.

## Introduction

Acute respiratory tract infections (ARTI) are one of the most important causes of morbidity and mortality in children; they are estimated to be responsible for approximately two million childhood deaths globally.^1–3^ The most common etiological agents causing ARTI in children are the respiratory viruses (about 80% of respiratory infections). Among the commonly encountered respiratory viruses, respiratory syncytial virus (RSV) has a tendency to cause severe respiratory tract manifestations, primarily bronchiolitis and pneumonia, among infants and young children. Bronchiolitis and pneumonia are the most common lower respiratory tract infections in children and the leading cause of hospital admission under six months of age. About 60–80% of all children hospitalized with bronchiolitis and pneumonia are infected by RSV.^4–7^ RSV accounts for about 33.1 million episodes of acute lower respiratory tract infections per year in children younger than five years, with 3.2 million cases leading to hospitalization and up to 118,200 deaths.^8^ It is important to note that there are links between RSV-associated bronchiolitis and pneumonia and later development of asthma and wheeze.^9,10^

RSV infection leads to infiltration of various immune cell types; if the lung recruitment of immune cells is dysregulated, the balance of viral control versus tissue damage is lost, resulting in pathology and severe disease.^11-13^ Despite many efforts to develop an RSV vaccine, there is a lack of effective prophylactic and therapeutic agents, even though research continues to uncover the complex mechanisms underlying RSV immunopathogenesis.^14^ Several factors have been linked to the severity of RSV infection in children, including virus-related factors such as viral types and genotypes and host-related factors such as preterm birth, birth defects, and genetic variations.^15-18^ Genetic analyses of children show an association of different polymorphisms in specific host genes with severe RSV infection.^19^

Cannabinoids refer to a group of diverse components that include substances of the plant *Cannabis sativa*, endocannabinoids, and synthetic ingredients.^20^ The biological effects of cannabinoids are mediated through the activation of G-protein-coupled cannabinoid receptors including CB1 and CB2.^21^ The psychoactive function of cannabinoids are mediated through CB1 as it is abundantly expressed in the central nervous system. High-level CB2 expressions by immune cells and the inducible expressions of these receptors in inflammatory condition suggest that the anti-inflammatory and immunomodulatory action of cannabinoids are CB2-dependent.^22^ This provides a rationale for a novel immunoregulatory role for the cannabinoid system as a target for drug discovery for the treatment of multiple inflammatory disorders.^21^ Recent studies have suggested a role for CB2 gene (CNR2) polymorphism in immunity-associated diseases pathogenesis.^23–25^ The single nucleotide polymorphism (SNP) rs35761398 in CNR2, which encodes the CB2 receptors, substitutes glutamine (Q) 63 with arginine (R) and affects the response of CB2 to cannabinoids. The receptor carrying R showed a reduced immune modulation function when activated by cannabinoids.^26^

Although the effects of the endocannabinoid system on immunity have received considerable attention, their impact on respiratory viral immunopathology are still unclear. It may be beneficial to explore the implication of the cannabinoid system and the virus may to maintain immune homeostasis in viral infections. In this regard, we studied the possible role of the CB2 Q63R functional variant in respiratory disease severity in children and the frequency distribution of RSV infection in children. We expanded our CB2 studies to an *in vivo* system in which CB2 was blocked by the selective antagonist AM630 during RSV infection. In addition, by using the CB2 selective agonist JWH133, we further tested how CB2 activation affects the clinical outcome of RSV infection. The results reported here provide evidence that CB2 receptors play an important role in RSV disease severity.

## Results

### Human studies

#### Clinical data

Between December 2015 and April 2016, we admitted 180 patients (90 inpatients and 90 outpatients) to the Bahrami Children Hospital. The patients' ages ranged from one month to 22 months with median and mean ages of three and 4.65 months respectively. The common clinical symptoms were sneezing, runny nose, cough, dyspnea, and fever. The details of demographic, age, gender, month, and clinical data according to the history of inpatients and outpatients are shown in Table 1. Of all the children enrolled in the study, 83 patients (46.1%) were found positive for RSV. The prevalence of RSV was 45/83 (54.2%) in inpatients and 38/83 (45.8%) in outpatients. Males (57.8%) were more affected than females among the RSV-positive patients. The highest rates of RSV infection were detected during winter.

**Table 1.** Demographic, gender, age, month, and clinical data according to inpatients and outpatients.

		All cases			RSV (+) cases		
		Inpatient	Outpatient	Total	Inpatient	Outpatient	Total
**Demographic, gender, age, month, and clinical data according to inpatients and outpatients (n (%))**	**Age (month)**						
	Median	3	3	3	3	3	3
	Mean	4.78	4.51	4.65	4.44	4.02	4.23
	**Sex**						
	Male	52 (57.8)	48 (53.3)	100 (55.6)	25 (55.6)	23 (60.5)	48 (57.8)
	Female	38 (42.2)	42 (46.6)	80 (44.4)	20 (44.4)	15 (39.5)	35 (42.2)
	Total	90 (100)	90 (100)	180 (100)	45 (100)	38 (100)	83 (100)
	**Months**						
	December 2015	3 (3.3)	1 (1.1)	4 (2.2)	3 (6.7)	1 (2.6)	4 (4.81)
	January 2016	65 (72.2)	46 (51.1)	111 (61.7)	33 (73.3)	20 (52.7)	53 (63.86)
	February 2016	5 (5.6)	42 (46.7)	47 (26.1)	3 (6.7)	17 (44.7)	20 (24.1)
	March 2016	17 (18.9)	1 (1.1)	18 (10)	6 (13.3)	0 (0)	6 (7.23)
	Total	90 (100)	90 (100)	180 (100)	45 (100)	38 (100)	83 (100)
	**Age groups**						
	1–2 months	34 (37.8)	40 (44.4)	74 (41.1)	18 (40)	15 (39.5)	33 (39.8)
	2–3 months	13 (14.4)	8 (8.9)	21 (11.7)	8 (17.8)	5 (13.2)	13 (15.7)
	3–4 months	7 (7.8)	5 (5.6)	12 (6.7)	4 (8.9)	1 (2.6)	5 (6)
	4–5 months	4 (4.4)	9 (10)	13 (7.2)	3 (6.7)	6 (15.8)	9 (10.8)
	5–6 months	11 (12.2)	5 (5.6)	16 (8.9)	5 (11.1)	2 (5.3)	7 (8.4)
	6–8 months	6 (6.7)	11 (12.2)	17 (9.4)	2 (4.4)	6 (15.8)	8 (9.6)
	8–10 months	3 (3.3)	2 (2.2)	5 (2.8)	1 (2.2)	1 (2.6)	2 (2.4)
	10–12 months	7 (7.8)	8 (8.9)	15 (8.3)	3 (6.7)	1 (2.6)	4 (4.8)
	12–22 months	5 (5.6)	2 (2.2)	7 (3.9)	1 (2.2)	1 (2.6)	2 (2.4)
	Total	90 (100)	90 (100)	180 (100)	45 (100)	38 (100)	83 (100)
	**Clinical data**						
	Fever	43 (47.8)	23 (25.6)	66 (36.7)	24 (53.3)	11 (28.9)	35 (42.2)
	Sore throat	5 (5.6)	7 (7.8)	12 (6.7)	4 (8.9)	4 (10.5)	8 (9.6)
	Cough	83 (92.2)	88 (97.8)	171 (95)	42 (93.3)	38 (100)	80 (96.4)
	Dyspnea	73 (81.1)	52 (57.8)	125 (69.4)	36 (80)	21 (55.3)	57 (68.7)
	Runny nose	87 (96.7)	88 (97.8)	175 (97.2)	44 (9.8)	37 (97.4)	81 (97.6)
	Nasal congestion	69 (76.7)	77 (85.6)	146 (1.1)	36 (80)	34 (89.5)	70 (84.3)
	Restlessness	3 (3.3)	1 (1.1)	4 (2.2)	3 (6.7)	1 (2.6)	4 (4.8)
	Sneezing	84 (93.3)	88 (97.8)	172 (95.6)	41 (91.1)	37 (97.4)	78 (94)
	Hemoptysis	1 (1.1)	3 (3.3)	4 (2.2)	1 (2.2)	2 (5.3)	3 (3.6)
	Vomiting	3 (3.3)	0 (0)	3 (1.7)	3 (6.7)	0 (0)	3 (3.6)
	Chills	1 (1.1)	0 (0)	1 (0.6)	1 (2.2)	0 (0)	1 (1.2)
	Anorexia	2 (2.2)	0 (0)	2 (1.1)	2 (4.4)	0 (0)	2 (2.4)
	Pneumonia	53 (58.9)	58 (64.4)	111 (61.7)	29 (64.4)	22 (57.9)	51 (61.4)
	Bronchiolitis	6 (6.7)	0 (0)	6 (3.3)	4 (8.9)	0 (0)	4 (4.8)
	Suspected to RSV	32 (35.6)	28 (31.1)	60 (33.3)	17 (37.8)	14 (36.8)	31(37.3)

#### Genetic data

The genotype distribution and allele frequencies for the CB2 Q63R polymorphism of inpatients and outpatients with ARTI are shown in Table 2. The frequencies of CB2 Q63R polymorphisms were found in the Hardy–Weinberg equilibrium among all the patients (*p* = 0.97), inpatients (*p* = 0.43), and outpatients (*p* = 0.23). We found a significant difference between genotypic and allelic distributions of the Q63R polymorphism between the inpatients and outpatients. The R allele, a common allele of rs35761398, showed a lower frequency for hospitalized infants compared with controls (OR = 0.630, 95% CI: 0.408–0.0973; *p* = 0.037)-this mean that the common allele reduced the risk of hospitalization. Interestingly, a significant overrepresentation of the QQ genotype and the Q allele was observed in the hospitalized patients compared with controls (OR = 3.275, 95% CI: 1.221–8.705; p = 0.019 and OR = 1.586, 95% CI: 1.027–2.449; p = 0.037 respectively). The relative odds ratio suggested that the risk of hospitalization was more than three-fold in children with the QQ genotype.

**Table 2.** Genotype and allele frequencies of CNR2 gene in patients with acute respiratory infection.

			Inpatient	Outpatient	P-value	OR (95% CI)
**Genotype and allele frequencies of CNR2 gene in patients with acute respiratory infection (n (%))**	**All cases**	QQ	17 (73.9)	6 (26.1)		1
		QR	40 (48.25)	43 (51.8)	0.033	3.045 (1.092–8.493)
		RR	33 (44.6)	41 (54.4)	0.017	3.520 (1.247–9.934)
		QQ	17 (73.9)	6 (26.1)		1
		QR+RR	73 (46.5)	84 (53.5)	0.019	3.275 (1.221–8.705)
		RR	33 (44.6)	41 (55.4)		1
		QR+QQ	57 (53.8)	49 (46.2)	0.156	0.642 (0.381–1.256)
		Q	74 (57.4)	55 (42.6)		1
		R	106 (45.8)	125 (54.2)	0.037	1.586 (1.027–2.449)
		R	106 (45.8)	125 (54.2)		1
			Q	74 (57.4)	55 (42.6)	0.037	0.630 (0.408–0.0973)
	**RSV (+) cases**	QQ	9 (81.8)	2 (18.2)		1	
		QR	18 (56.2)	14 (43.8)	0.144	3.5 (0.649–18.852)	
		RR	18 (45)	22 (55)	0.043	5.5 (1.052–28.752)	
		QQ	9 (81.8)	2 (18.2)		1	
		QR+RR	36 (50)	36 (50)	0.065	4.5 (0.908–22.29)	
		RR	18 (45)	22 (55)		1	
		QR+QQ	27 (62.8)	16 (37.2)	0.106	0.48 (0.201–1.166)	
		Q	36 (66.6)	18 (33.4)		1	
		R	54 (48.2)	58 (51.8)	0.026	2.148 (1.092–4.224)	
		R	54 (48.2)	58 (51.8)		1	
		Q	36 (66.6)	18 (33.4)	0.026	0.465 (0.236–0.915)	

The same result was observed in RSV-positive inpatients and outpatients. Our data indicated that the polymorphisms of the CNR2 gene (Q63R) also affected the clinical course of RSV infection (Table 2). In RSV-infected children, the R allele had a lower frequency for inpatients than outpatients (OR = 0.465, 95% CI: 0.236–0.915; *p* = 0.026 respectively). Thus, the common allele is associated with a reduced risk of hospitalization in RSV-positive patients. Moreover, in case of RSV-infected patients, overrepresentation of the QQ genotype and of the Q allele was observed in inpatients compared with outpatients (OR = 4.5, 95% CI: 0.908–22.29; *p* = 0.065 and OR = 2.148, 95% CI: 1.092–4.224; *p* = 0.026, respectively). The associated risk of developing severe ARTI following RSV infection significantly increased more than two-fold in children carrying the Q allele. We did not find any association between CB2 Q63R variants and clinical features (Table 3).

**Table 3.** Demographic, gender, age, and clinical data according to CNR2 variants.

		All cases					RSV (+) cases				
		QQ	QR	RR	Total	P	QQ	QR	RR	Total	P
**Demographic, gender, age, month, and clinical data according to CNR2 variants (n (%))**	**Sex**										
	Male	11 (11)	41 (41)	48 (48)	100 (100)	0.109	5 (10.4)	16 (33.3)	27 (56.3)	48 (100)	0.220
	Female	12 (15)	42 (52.5)	26 (32.5)	80 (100)		6 (17.1)	16 (45.7)	13 (37.2)	35 (100)	
	Total	23 (12.7)	83 (46.1)	74 (41.2)	180 (100)		11(13.2)	32 (38.6)	40 (48.2)	83 (100)	
	**Age groups**										
	1–2 months	7 (9.45)	42 (56.75)	25 (33.8)	74 (100)	0.053	4 (12.1)	15 (45.4)	14 (42.5)	33 (100)	0.800
	2–3 months	3 (14.3)	9 (42.85)	9 (42.85)	21 (100)		2 (15.4)	6 (46.1)	5 (38.5)	13 (100)	
	3–4 months	2 (16.6)	4 (33.3)	6 (50)	12 (100)		2 (40)	1 (20)	2 (40)	5 (100)	
	4–5 months	4 (30.76)	5 (38.46)	4 (30.76)	13 (100)		1 (11.1)	2 (22.2)	6 (66.6)	9 (100)	
	5–6 months	0 (0)	7 (43.7)	9 (56.3)	16 (100)		1 (14.3)	1 (14.3)	5 (71.4)	7 (100)	
	6–8 months	0 (0)	5 (29.5)	12 (70.5)	17 (100)		1 (33.3)	1 (33.3)	1 (33.3)	3 (100)	
	8–10 months	0 (0)	3 (60)	2 (40)	5 (100)		0 (0)	1 (50)	1 (50)	2 (100)	
	10–12 months	5 (33.3)	5 (33.3)	5 (33.3)	15 (100)		0 (0)	2 (50)	2 (50)	4 (100)	
	12–22 months	2 (28.57)	3 (42.86)	2 (28.57)	7 (100)		0 (0)	0 (0)	2 (100)	2 (100)	
	Total	23 (12.8)	83 (46.1)	74 (41.1)	180 (100)		11(13.2)	32 (38.6)	40 (48.2)	83 (100)	
	**Clinical data**										
	Fever	7 (10.6)	31 (45)	28 (42.4)	66 (100)	0.801	5 (14.3)	13 (37.1)	17 (48.6)	35 (100)	0.960
	Sore throat	3 (25)	5 (41.7)	4 (33.3)	12 (100)	0.417	1 (12.5)	4 (50)	3 (37.5)	8 (100)	0.773
	Cough	21 (12.3)	79 (46.2)	71 (41.5)	171 (100)	0.668	10(12.5)	31 (38.7)	39 (48.8)	80 (100)	0.574
	Dyspnea	16 (12.8)	61 (48.8)	48 (38.4)	125 (100)	0.503	7 (12.3)	24 (42.1)	26 (45.6)	57 (100)	0.614
	Runny nose	22 (12.6)	80 (45.7)	73 (41.7)	175 (100)	0.612	11(13.6)	30 (37)	40 (49.4)	81 (100)	0.195
	Nasal congestion	19 (13)	63 (43.1)	64 (43.9)	146 (100)	0.235	9 (12.8)	26 (37.2)	35 (50)	70 (100)	0.746
	Restlessness	1 (25)	1 (25)	2 (50)	4 (100)	0.621	1 (25)	1 (25)	2 (50)	4 (100)	0.726
	Sneezing	22 (12.7)	77 (44.7)	73 (42.4)	172 (100)	0.204	11(14.2)	28 (35.8)	39 (50)	78 (100)	0.139
	Hemoptysis	0 (0)	2 (50)	2 (50)	4 (100)	0.735	0 (0)	2 (66.6)	1 (33.3)	3 (100)	0.551
	Vomiting	1 (33.3)	1 (33.3)	1 (33.3)	3 (100)	0.559	1 (33.3)	1 (33.3)	1 (33.3)	3 (100)	0.574
	Anorexia	1 (50)	1 (50)	0 (0)	2 (100)	0.220	1 (50)	1 (50)	0 (0)	2 (100)	0.207
	Pneumonia	17 (15.4)	54 (48.6)	40 (36)	111 (100)	0.159	8 (15.7)	19 (37.3)	24 (47)	51 (100)	0.710
	Bronchiolitis	1 (16.6)	4 (66.6)	1 (16.6)	6 (100)	0.462	1 (25)	3 (75)	0 (0)	4 (100)	0.142
	Suspected to RSV	6 (10)	24 (40)	30 (50)	60 (100)	0.223	4 (13.3)	11 (36.6)	15 (50)	30 (100)	0.963

### Mouse studies

#### The CB2 expression during primary RSV infection

**Figure 1.**
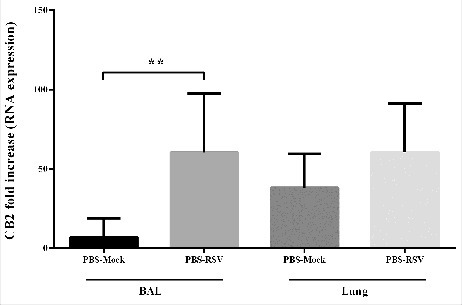
The Effect of RSV infection on CB2 receptors expression. Relative expression of CB2 receptor was evaluated in BAL and lung of mice, 5 day after primary RSV or mock infection, using specific primers targeting the CB2 genes, and normalized to those of the housekeeping gene (β-actin). Results represent the mean ±SEM of 6 animals for each group. (**p < 0.007).

To examine the effect of RSV infection on the expression of CB2 receptors, we measured the CB2 mRNA expression in the bronchoalveolar lavage (BAL) and the mice lungs five days after the viral challenge. Our results showed that the CB2 expression was significantly enhanced in the BAL cells of mice following RSV infection compared with uninfected mice (*p* < 0.007). We found no significant difference in the amount of the CB2 expression between the lung cells of infected and uninfected mice (Fig. 1).

#### CB2 blockade and activation during RSV infection

**Figure 2.**
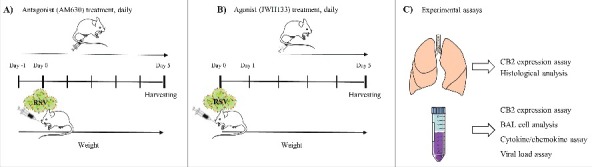
Experimental design. Time course of antagonist (A) and agonist treatment (B) and experimental assays (C) in RSV challenged mice.

**Figure 3.**
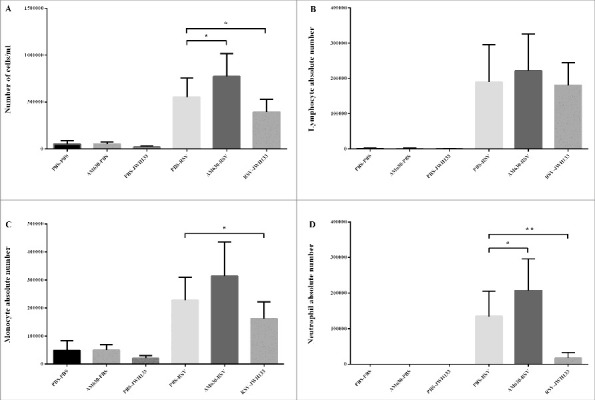
The Effect of CB2 receptors on immune cells influx following RSV infection. CB2 receptors were blockade via using AM630 or activated through JWH133 daily and the total cell counts (A), number of lymphocytes (B), monocytes (C), and neutrophil (D) of BAL cells were determined on day 5 after infection. Results represent the mean ±SEM of 6 animals for each group. (*p < 0.05, **p < 0.006).

The experimental design and the time course of antagonist (AM630) and agonist (JWH133) treatments in RSV-challenged mice are shown in Fig. 2. Primary RSV infection elicits an inflammatory response comprising a mixed population of leukocytes that infiltrates the pulmonary airways. The level of cells infiltration following RSV infection was significantly enhanced in the CB2 blockade via AM630 (*p* < 0.05) (Fig. 3). The differential analysis of the leukocytes in the BAL of mice showed that the CB2 blockade enhanced all leukocyte subsets, although only the neutrophil count was statistically significant (*p* < 0.05). In contrast, the data showed that CB2 activation through JWH133 significantly abrogated the RSV-induced leukocyte migration into the lungs five days after viral challenge (*p* < 0.05). The differential analysis of the leukocytes in the BAL of mice showed that CB2 activation following RSV infection significantly diminished neutrophils and monocytes counts (*p* < 0.006 and *p* < 0.05, respectively) (Fig. 3).

**Figure 4.**
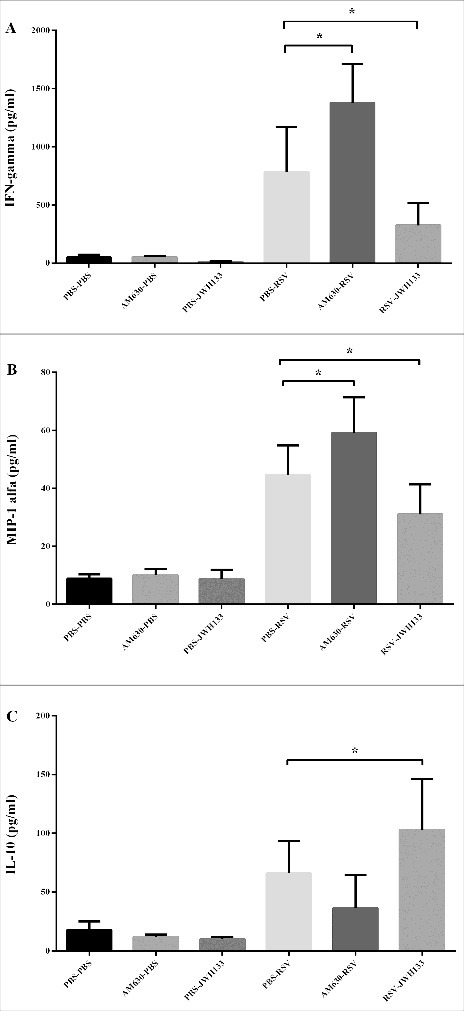
The Effect of CB2 receptors on cytokine/chemokine production following RSV infection. CB2 receptors were blockade via using AM630 or activated through JWH133 daily and the IFN-γ (A), MIP-1α (B) and IL-10 (C) production were determined in BAL supernatant on day 5 after infection. Results represent the mean ±SEM of 6 animals for each group. (*p < 0.05).

The secreted inflammatory and anti-inflammatory mediators were measured as indicators of immune cell function in response to RSV challenge. As compared with control conditions, the CB2 blockade significantly induced IFN-γ and MIP-1α production (*p*< 0.05) as well as non-significantly decreased anti-inflammatory cytokine IL-10 levels following RSV infection. While RSV infection significantly induced IFN-γ and MIP-1α and decreased IL-10 production, CB2 activation displayed significantly decreased concentrations of IFN-γ and MIP-1α and increased IL-10 concentrations in the BAL of mice five days after the viral challenge (*p* < 0.05) (Fig. 4).

**Figure 5.**
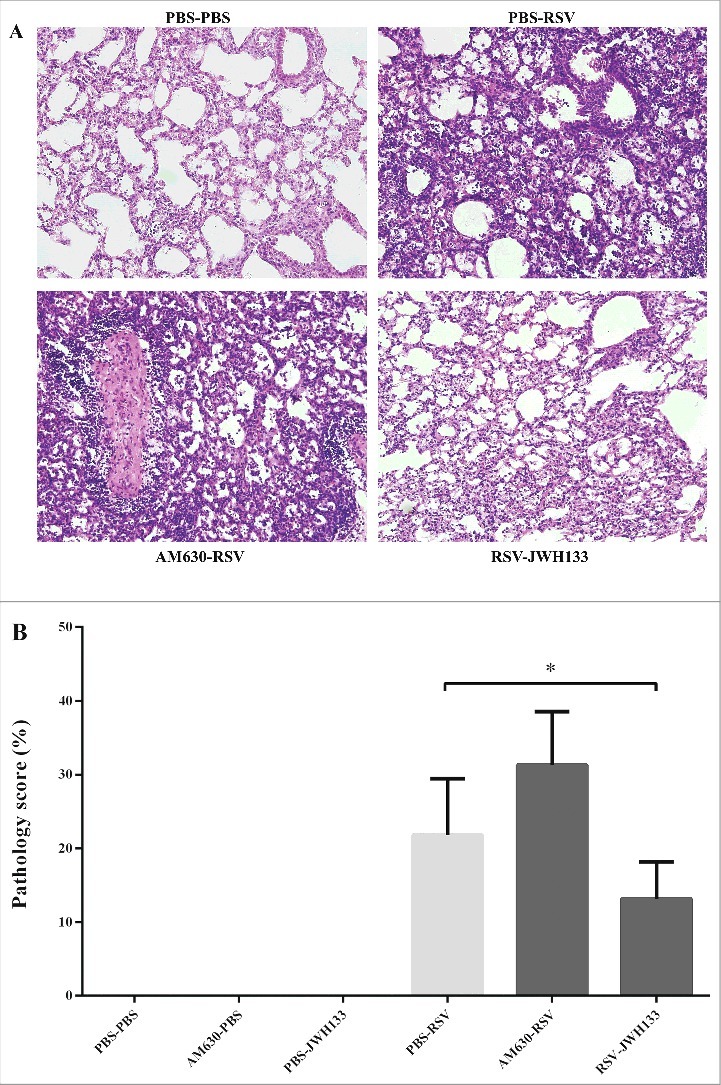
The effect of CB2 receptors on lung pathology following RSV infection. CB2 receptors were blockade via using AM630 or activated through JWH133 daily and the lung pathology were determined on day 5 after infection. (A) Representative slides of hematoxylin and eosin-stained lungs were analyzed and scored on day 5 after infection. (B) Pathology scores percentage for each group are shown. Results represent the mean ±SEM of 6 animals for each group. (*p < 0.05).

**Figure 6.**
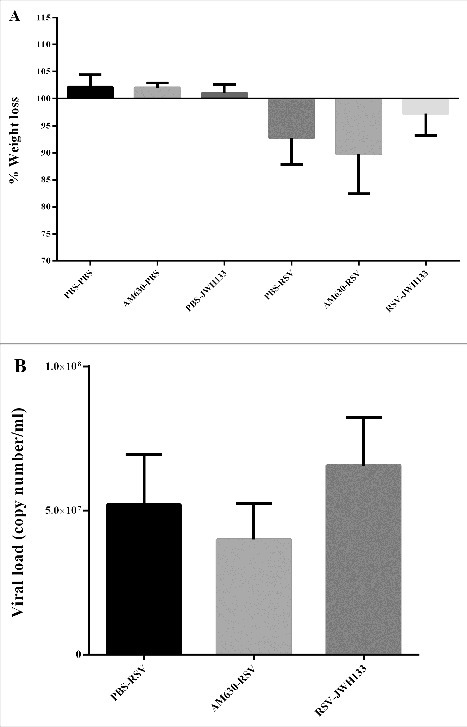
The effect of CB2 receptors on mice weight loss and viral load following RSV infection. (A) The graph shows changes in body weight 5 days after the primary RSV or mock infection. (B) CB2 receptors were blockade via using AM630 or activated through JWH133 daily and RSV copy number was determined by quantitative real-time polymerase chain reaction in BAL supernatant on day 5 after infection. Results represent the mean ±SEM of 6 animals for each group.

In agreement with the influx of BAL immune cells, the CB2 blockade impacts the accumulation of immune cells in the peribronchial and perivascular spaces of the lungs, although the differences did not reach statistical significance. In contrast, CB2 activation significantly decreased the accumulation of immune cells in the peribronchial and perivascular spaces of the lung following RSV infection (*p* < 0.05) (Fig. 5). In this experiment, we followed weight loss to determine whether the CB2 blockade and activation have any impact on disease severity. Weight loss is the hallmark of disease severity during RSV infection in the mice model. Our results showed that while the CB2 blockade is associated with weight loss after RSV infection, CB2 activation restored weight loss after RSV infection (Fig. 6A). Furthermore, our results did not show that the CB2 blockade and activation impacted viral replication (Fig. 6B).

In these experiments, no differences were found in mice that received PBS—whether they were treated with AM630 or JWH133 alone—i.e., antagonist and agonist induced no effects in the absence of RSV infection.

## Discussion

The detection of specific viral causes of infection provides a useful starting point for understanding illnesses attributable to ARTIs and will guide future research studies. Epidemiological studies on the prevalence of RSV infection are essential for developing diagnostic methods, efficient drugs, and vaccine design.^16^ Studies from Middle Eastern countries, such as Iran, are rare. The frequency distribution of RSV infection in our study was 46.1%. The RSV positivity rate was higher among inpatient children. The majority of RSV-positive children (75.6%) were between one to six months are in line with previous observations.^27^ Indeed, RSV represents a substantial burden of ARTIs particularly in very young children, leading to severe morbidity and hospitalization.^28^ Even with a limited sample size, this study still provides much-needed information on the prevalence of RSV in children aged less than two years in the largest city of Iran. Significant peaks of RSV prevalence were detected in the winter. This finding underlined the seasonal characteristics of RSV infections in our study group—this was consistent with the age patterns and seasonal RSV prevalence reported in other studies.^29^ Seasonality of virus is an important factor to optimize the timing for potential interventions against these diseases and early treatment therapies. The present study primarily focuses on RSV infection as the main cause of severe respiratory tract manifestations in children, while the possibility of other viral respiratory infections, such as rhinovirus, metapenumovirus, parainfluenza virus, and boca virus, needs further studies.

Our knowledge of RSV pathogenesis and disease severity has increased over the last few years. It is crucial to understand how RSV interacts with its host to facilitate the development of safe and effective therapeutic interventions.^30,31^ Genetic analyses are not only important in showing the likelihood of risk development toward severe infections, but also provide information that characterizes the disease pathogenesis.^32^ We had previously shown that genetic variations in the opioid system (OPRM1), which acts like a cannabinoid system, is associated with RSV disease severity: Carriers of the G allele are less prone to develop severe RSV infections than those carrying the A allele.^19^ The CB2 variants have been shown to affect the ability of the CB2 receptor differently to exert its function.^26^ The CB2 Q63R variant affects the response of CB2 to cannabinoid by differently modulating the endocannabinoid-induced immune hemostasis, and consequently, results in a lack of balance for immune responses that may turn out to be a possible risk for immunity-associated disorders.^33^ The rationale of our study is based on the assessed association between the CB2 Q63R variant and several immunity-associated disorders as well as on the well-known anti-inflammatory and immunomodulatory effects of CB2 signaling.^23–25^

The data from our study, for the first time, suggests that those with the CB2-63 RR variant is less prone to developing severe respiratory tract infections and those with the CB2-63 QQ variant is associated with more severe respiratory tract diseases and the risk of hospitalization. Although the study does not consider all respiratory viruses, the same result was observed in RSV-positive infants carrying the Q allele. An explanation for the association between the CB2 Q63R variant and the risk of disease severity and hospitalization in children with acute respiratory tract infections may be that the immune response with QQ variants were more inhibited when activated by an endogenous cannabinoid—i.e. a less aggressive immune response against the virus—thereby allowing the virus to replicate and induce severe infection. Sip et al. found that the CB2-mediated inhibition of lymphocyte proliferation deriving from QQ subjects was normal, while the one derived from RR subjects was reduced.^33^ Such data indicated that the CB2 Q63R variant will be part of the disease severity caused by RSV, but further clinical investigations for larger case studies are needed to confirm this.

In the animal study, we found that RSV infection can affect the CB2 expression in BAL cells—this is in line with its high-level expression by immune cells and the inducible expression in inflammatory condition.^22^ In this study, the CB2 blockade is associated with enhanced immunopathology, such as immune cells influx and increased IFN-γ and MIP-1α production, along with RSV-induced lung pathology and weight loss. The results of our study are in line with previous reports on the role of cannabinoids in influenza infection outcomes.^34,35^ Buchweitz et al. and Karmaus et al. have shown that cannabinoid receptors null mice exhibit a significantly enhanced inflammatory response to influenza infection, thereby strongly suggesting an endogenous role for cannabinoid receptors in immune regulation.^34,35^ Taken together, the current study and those reported by others suggest that the CB2 receptors, as the primary signaling pathway for endocannabinoid immune modulation, may play a crucial role in maintaining immune homeostasis and controlling the magnitude of the immune response through negative regulation. However, to replicate the RSV infection seen in children with different disease severity in a better way, various RSV challenge doses should be tested in future studies.

Since the activation of CB2 triggers anti-inflammatory action, targeting these receptors may be a novel and effective approach for the treatment of RSV-associated immunopathology.^30^ In the present work, CB2 activation enhanced IL-10 production and reduced bronchoalveolar cellular influx and IFN-γ and MIP-1α production. These effects result in the control of RSV-induced lung pathology and weight loss. This observation was consistent with the immunosuppressive effects of CB2 activation reported by others.^36–41^ Some studies previously indicated that CB2 activation inhibits immune cells recruitment by activating the MAPK pathway.^37,38^ Correa et al. have shown that JWH-133 induces a sustained activation of ERK1/2 MAP kinase, thereby contributing to the downregulation of inflammatory cytokines.^38^ According to Kurihara et al., CB2 activation plays a role in regulating excessive inflammatory response by controlling RhoA activation.^42^ Moreover, JWH-133 ameliorates chronic colitis by inducing apoptosis, reducing the number of activated T cells, suppressing mast cells, NK cells, and neutrophils at inflammation sites.^43^ One of the main findings in the present study was that JWH133 significantly decreases neutrophils recruitment following RSV infection. Neutrophils play an important role in the immunopathogenesis of RSV infection.^44^ Our results are in line with Krohn et al. and Andrade-Silva et al., who reported that CB2 activation inhibits neutrophil recruitment.^45,46^ Murikinati et al. detected that JWH133 administration inhibits neutrophil recruitment at the site of inflammation by activating p38.^37^ Thus, our results imply that CB2 activation may act as a novel immunomodulatory strategy to alleviate RSV-induced lung pathology through the inhibition of immune cells. However, it is important to ensure that the JWH133 effects are attenuated with AM630 in future studies.

In conclusion, given the breadth of cannabinoids-mediated regulation of the immunity function and the complicated immunopathology associated with RSV infection, our results indicate that: (i) the CB2 Q63R variant is associated with the clinical course of acute respiratory viral infections (ii) experimental RSV-induced immunopathogenesis can be modulated in part by endocannabinoids, as observed by blocking the CB2 signaling, and (iii) RSV-induced immunopathogenesis can be modulated by CB2 activation and may be a novel approach for the treatment of RSV bronchiolitis in children. However, caution is required due to beneficial/harmful functions of endocannabinoid systems. It is important to note that different phases/stage of RSV infection and different age groups may require different treatment approaches.

## Patients and methods

### Patients

We recruited 180 Iranian children under two years of age with clinically suspected acute respiratory viral infection, including 90 hospitalized patients (severe ARTI) and 90 outpatients (mild ARTI) during the winter season of 2016 at Bahrami Children's Hospital, Tehran, Iran. According to the Bahrami Children's Hospital protocol used for treatment of severe ARTI; the hospitalization criteria contain respiratory distress including tachypnea, nasal flaring, chest retractions, or grunting. The respiratory rate >60 for children less than 2 month, respiratory rate >50 for children between 2 month to 1 year and respiratory rate >40 for children between 1 to 3 years old were considered for hospitalized patients. A clinical questionnaire was used to collect data from all patients including age, gender, and symptoms like bronchiolitis, pneumonia, fever, sore throat, cough, dyspnea, runny nose, nasal congestion, and sneezing. The current human study was approved by science and bioethics committee of Tehran University of Medical Sciences.

### Genotype analysis

Genomic DNA was extracted from nasopharyngeal swab samples using a DNA extraction kit following manufacturers' instructions (Roche, Germany). All samples were genotyped for the CNR2 rs35761398 variant, which changes the second and third adenosine at codon 63 (CAA) to guanosine (CGG) leading to the missense variant Q63R in the first intracellular signaling loop of the encoded CB2 protein. The genotypes of Q63R variation were determined by using a TaqMan assay with commercial probes (Thermo Fisher, USA). Both PCR and post-PCR allelic discrimination was performed on an ABI PRISM 7900 system (Applied Biosystems, USA). Genotypes of random samples (10% in each experimental group) were confirmed by direct PCR sequencing with 5′-ATGCTGGGTGACAGAGATAG-3′ (F), and 5′-AGTCACGCTGCCAATCTTCA-3′ (R) primers. The reaction conditions were as follows: 94°C for 4 min followed by 30 cycles of 94°C for 50 s, 55°C for 45 s, and 72°C for 50 s.

### Virus detection

Viral RNA was extracted from nasopharyngeal swab samples using viral high pure nucleic acid extraction kit following manufacturers' instructions (Roche, Germany). Extracted RNA was converted into cDNA using a high-capacity cDNA Reverse Transcription kit (Applied Biosystems, USA) according to the manufacturer's instructions. The presence of RSV genome (N gene) in all collected samples was analyzed through conventional PCR with 5′-GGAACAAGTTGTTGAGGTTTATGAATATGC-3′ (F), and 5′-TTCTGCTGTCAAGTCTAGTACACTGTAGT-3′ (R) primers. The reaction conditions were as follows: 94°C for 4 min followed by 40 cycles of 94°C for 30 s, 55°C for 30 s, and 72°C for 1 min.

### Animal experiment

Six to nine-week-old female Balb/c mice (n = 36, 6 mice/group), weighing 15–18 g were obtained from the Institute Pasteur, Karaj, Iran. The animals were transferred and maintained in their home cages one week before the beginning of the experiments. Mice were maintained in individual cages with food/water *ad libitum* and in a controlled environment in the animal house of Tehran University of Medical Sciences. All animal experiments were approved by the animal ethics committee of the Tehran University of Medical Sciences.

Mice were randomly assigned into controls (PBS-PBS, AM630-PBS, PBS-JWH133) and challenged (PBS-RSV, AM630-RSV, RSV-JWH13) groups. In order to investigate the role of CB2 receptors in the immunopathogenesis of RSV infection, AM630 (Sigma, USA) was injected i.p. twice a day at a dose of 2 mg/kg. One day after the first antagonist treatment, mice were anesthetized with ketamine/xylazine and intranasally challenged with RSV-A2 at 5 × 10^6^ pfu/50 µl/mice dose. To determine the potential therapeutic effects of CB2 receptors activation in the course of RSV infection, JWH-133 (Tocris Cookson Ltd, U.K.) was injected i.p. 24 hrs after viral challenge (5 × 10^6^ pfu/50 µl/mice) twice a day at a dose of 5 mg/kg. The antagonist and agonist injection were continued daily up to 5 days after infection and mice were sacrificed at day 5, the peak day of viral load and inflammatory cells influx into the airways, and BAL fluid (BALF) and lungs were obtained, and then CB2 receptors expression, airway immune cells influx, cytokine/chemokine secretion, lung histopathology, and viral load were assayed.

Drugs were dissolved in dimethyl sulfoxide and diluted in normal saline 0.9% before administration. Virus stuck was propagated on HEp-2 cells and purification was performed using polyethylene glycol precipitation as described previously by our group.^19^ The purified virus was resuspended and administrated in phosphate-buffered saline (PBS) and the control groups received a similar volume of PBS.

### CB2 receptors expression

In order to determine the expression level of CB2 receptors following RSV infection, total RNA was extracted from BAL cells and homogenized left lung lobe using RNX reagent (SinaClone, Iran), according to the manufacturer's instructions. Extracted mRNA was converted into cDNA using 1 µg total RNA in a 25 µl reaction volume with a high-capacity cDNA Reverse Transcription kit (Applied Biosystems, USA) according to the manufacturer's instructions. For CB2 gene expression, Real-time RT-PCR was performed on an ABI PRISM 7900 sequence-detection system (Applied Biosystems, USA) using the SYBRs Premix ExTapTM II (Takara, Japan). The reaction conditions were as follows: 95°C for 30 s, followed by 40 cycles of 95°C for 5 s, 57°C for 30 s, and 60°C for 30 s. Melting curve conditions were as follows: 95°C for 15 s and 65°C for 1 min. The relative level of gene expression was determined by the comparative threshold cycle method as described by the manufacturer. Level of CB2 gene expression was normalized to those of the housekeeping gene (β-actin) using the 2^−ΔΔCt^ method and expressed in the graphs as “relative expression”. The following primer pairs were used: CB2: 5′-GGTGGACTTGTTGCCCTAGT-3′ (F), 5′-TAGAAGCCAGCCCAGTAGGT-3′ (R), and β-actin: 5′-GCTCTGGCTCCTAGCACCAT-3′ (F), 5′-GCCACCGATCCACACAGAGT-3′ (R).

### BAL fluid cell analysis

BALF was obtained 5 days after infection as described previously by our group.^19^ The total number of cells present in the BALF was counted with the aid of a light microscope in Neubauer chambers, and differential leukocyte counts were performed on smears stained with Giemsa dye.

### Cytokine and chemokine assay

The secretion of IFN-γ, MIP-1α and IL-10 were measured with ELISA kits (PeproTech, USA), according to the instructions of the manufacturer. Concentrations of cytokines in the samples were calculated by interpolation from the standard curve. The threshold of sensitivity for IFN-γ, MIP-1α, and IL-10 were 16 pg/ml, 4 pg/ml, and 39 pg/ml, respectively.

### Histological analysis

Five day after challenge, mice were sacrificed and their lung was obtained, and histology slides were prepared as described previously by our group.^19^ Prepared slides were evaluated by light microscope and lung pathology, peribronchial and perivascular infiltration in the lungs were scored as described previously.^47,48^ The average of the sum of each reading was compared among groups.

### Viral load assay

Nucleic acid was prepared from supernatants of BALF using viral high pure nucleic acid extraction kit following manufacturers' instructions (Roche, Germany). Real-time RT-PCR was performed on an ABI PRISM 7900 sequence-detection system (Applied Biosystems, USA) using TagMan PCR Master Mix (Primer Design, UK) and results were expressed as copy number/ml as described previously.^19^

### Statistical analyses

To evaluate the significance of differences in genotype and allelic frequencies a chi-square test or Fisher's test was performed by using SPSS 23 software (IBM, Chicago, IL). Ninety-five percent confidence interval (95% CI) and Odds ratio (OR) were calculated. The OR was adjusted by age and sex in the logistic regression model. A linear logistic regression was performed to analyze clinical data with respect to the CB2 Q63R variant.

Graph preparation and statistical analyses in animal study were performed using GraphPad Prism v6.0 for Windows (GraphPad Software Inc., San Diego, CA, USA). The mean ±SEM is expressed in all graphs. The normality of date was performed using Kolmogorov–Smirnov test. Differences between two groups were carried out using Student's t test for unpaired data. In both human and animal study, differences were considered significant for *p* values < 0.05.

## Abbreviations

ARTI: Acute respiratory tract infections
RSV: Respiratory syncytial virus
SNP: Single-nucleotide polymorphism
OR: Odds ratio
CI: Confidence interval
BAL: Bronchoalveolar lavage
IFN-γ: Interferon-gamma
MIP-1α: Macrophage inflammatory protein 1-alpha
IL-10: Interleukine-10
BALF: BAL fluid
HEp-2: Epithelial type 2
PBS: Phosphate buffer saline
